# The objective structured clinical examination revisited for postgraduate trainees in general practice

**DOI:** 10.5116/ijme.52eb.f882

**Published:** 2014-03-04

**Authors:** Birgitte Schoenmakers, Johan Wens

**Affiliations:** 1 Academic Centre of General Practice, Department of Public Health and Primary Care, University of Leuven, Belgium; 2 Department of Public Health and Primary Care, Campus Drie Eiken, University of Antwerp, Belgium

**Keywords:** OSCE, postgraduate medical trainees, general practice, assessment

## Abstract

**Objectives:**

To investigate if the psychometric qualities of an OSCE consisting of more complex simulated patient encounters remain valid and reliable in the assessment of postgraduate trainees in general practice.

**Methods:**

In this intervention study without control group, the traditional OSCE was formally replaced by the new, complex version. The study population was composed by all postgraduate trainees (second and third phase) in general practice during the ongoing academic year. Data were handled and collected as part of the formal assessment program. Univariate analyses, the variance of scores and multivariate analyses were performed to assess the test qualities.

**Results:**

A total of 340 students participated. Average final scores were slightly higher for third-phase students (t-test, p=0.05). Overall test scores were equally distributed on station level, circuit level and phase level. A multiple regression analysis revealed that test scores were dependent on the stations and circuits, but not on the master phase.

**Conclusions:**

In a changing learning environment, assessment and evaluation strategies require reorientation. The reliability and validity of the OSCE remain subject to discussion. In particular, when it comes to content and design, the traditional OSCE might underestimate the performance level of postgraduate trainees in general practice. A reshaping of this OSCE to a more sophisticated design with more complex patient encounters appears to restore the validity of the test results.

## Introduction

The objective structured clinical examination (OSCE) has been used since the early 1970s to assess undergraduate and graduate medical students’ skills and competence in simulated patient encounters.^1^-^3^ Although intensively debated, the test quality regarding validity and reliability requires continual attention. In particular, worries arose regarding the capacity of the test outcome.^4-6^ This test quality is determined by the test content, test setting, and target population (students). Features such as test content and test setting are controllable and modifiable, but must address the target students. With respect to the workplace- based learning concept and in agreement with the learning objectives, the undergraduate OSCE will therefore focus on basic skills (practical and technical) with or without simulated patient contact. Further on in the curriculum, the OSCE scope will turn to more comprehensive patient encounters and addressing specific tasks (e.g., diagnosis, history taking, communication).^7,8^

In a setting in which educational concepts are rapidly changing, a revisiting of the OSCE should be considered. ^9^In general, students are better educated, higher skilled, and (fairly importantly) more confident with test and assessment proceedings. First, the concepts of workplace-based learning and the more sophisticated version termed ‘real- life learning’ penetrated the curricula.^10,11^ Second, many contemporary curricula provide comprehensive testing, feedback, and remediation programs for every student.^12,13^

Parallel to these changes in the educational environment, the assessment strategies and proceedings need to be reshaped.^14^

Given this point of view, the OSCE might benefit from a remodeling for postgraduate trainees in general practice. The content reliability of the OSCE when addressing this particular student population is a topic of discussion at the four Flemish Universities (Belgium). Specifically, in the past three academic years, mean OSCE scores rose remarkably (up to 75/100). This observation coincided with preserved psychometric qualities, which were expressed as reliability and validity. After meticulous revision of the past OSCEs, the most feasible explanation appeared to be that the test content had been insufficiently adjusted to the qualification level of these ‘professional students’.^15^ In other words, at this stage trainees were confronted with the more complex reality of consultation, while the OSCE focused on single aspects of patient encounters.

This pilot study addressed the following research question: do the psychometric qualities of an OSCE consist- ing of more complex simulated patient encounters remain valid and reliable during the assessment of postgraduate trainees in general practice? To provide complexity to the encounters, the OSCE introduced more than one focus, addressed more than one theme, and formulated more than one instruction per station.

## Methods

### Study population

The study population consisted of the second- and third- phase postgraduate trainees in general practice at the four Flemish Universities (University of Gent, University of Antwerp, University of Brussels, and University of Leuven, n=340). The GP-master comprises 3 years of a combined internship and academic education. Evidently, there were no exclusion criteria. Participation in the OSCE is mandatory, as it is part of the formal program assessment. However, postgraduate trainees that pass the OSCE during the second phase are rewarded with an exemption from this exam during the third phase.

### Ethical approval

The study was officially approved by the Medical Education Advisory Board, which is composed of students, teachers, and the heads of department of all four universities involved. Ethical approval is granted by the Medical Ethical Advisory Board of all four universities for educational research during the ongoing academic year. Following the national legislation, informed consent of the study population is only required when patients are involved.

### Proceedings of the OSCE development

The traditional OSCE was formally replaced by the new, complex version. This alteration was officially approved by the Medical Education Advisory Board, which is composed of students, teachers, and the heads of department of all four universities. Students were officially informed about the proceedings and about the changes in exam structure and content.

The development of the complex OSCE was preceded by debates with experts, students, and teachers. Above, a comprehensive analysis of the OSCEs from the past 10 years and a review of literature were conducted. A new protocol was developed that contained script conditions and instructions for the observer and simulated patient training. The blueprint of the OSCE was framed with respect to the learning objectives of the GP-master. Four experienced script-writers (all PhD-qualified teachers and practicing GPs) each produced eight stations in deliberation with their assistants and staff members. During the next stages, all OSCE scripts repeatedly passed the review and revision process. In total, 32 scenarios were written. The complexity of the OSCE was defined as the integration of two or more skills in one station. This approach more closely reflects a realistic situation, while preserving the structured and standardized character of the assessment.

### Scenario

Each scenario was constructed to embody two reasons for the encounter, instead of a single reason (as in a traditional station). Both reasons were content-related to avoid unnecessary confusion and pitfalls. For example: ‘a father has been referred to a GP by the school doctor of his 6-year-old son. The boy is overweight. Aside from this announcement, the father has some questions based upon irrational beliefs about the boy’s flat feet, assuming that this condition requires treatment before the boy can begin to participate in sports’.

### Simulated patient

The simulated patient (SP) was instructed to spontaneously mention the first reason for encounter. When students asked whether there were any other concerns, the simulated patient released additional information and disclosed the second (related) reason for encounter. To avoid the post- graduate trainee completely missing this second concern (and undeservedly failing this station), the SP was instructed to release the necessary information after a certain period of time. Students who did not spontaneously elicit both announcements were penalized on the item checklist (item: ‘student spontaneously elicits second announcement’).

### Candidate

Consequently, the instruction to the student was multifaceted. Students were asked to address two or more issues, for example: ‘take the anamnesis, discuss your findings, and give information’. Supplementary information about the clinical examination, lab results, medical history, etc. was available if required. This option guaranteed that the OSCE retained it structured and standardized character.

### Observers

Observers were accurately informed in advance. Prior to their actual involvement, they received a document in which the test-outcome problem was illustrated and the modification of the OSCE was explained and justified. Observers participated in group training just before the start of the OSCE. They were informed about assessment strategies and the general test-outcome, their task as the observer, the construction of the OSCE script, and the scoring system. To set the standard for each station, the observers were asked to score each student’s performance independent of the item checklist (borderline regression method). To estimate the inter-rater reliability, certain stations were observed by two independently scoring observers.

### Scoring

The item checklist was accompanied by legends. The legends contained scoring instructions, illustration of skills, competencies, justification of items, etc. This option reduced the risk of discussion and inter-rater variability and enhanced the observers’ examination competence. Although the contribution of each item on the checklist to the maximum test score was weighted, observers were blinded for this intervention. Observers were also asked to assign a single score for each student’s performance on the station. This score was used to set standards (borderline regression method).

### Analysis

Results were analyzed with the aid of SAS version 9.1.3 and Excel 2010. Univariate analyses were performed to manage and explore the data (PROC UNIVARIATE in SAS). We calculated the variance of score in between the two master phases, in between stations, and in between circuits. The circuits varied across the different sessions, requiring a multivariate analytic approach to assess test qualities. Cronbach’s alpha was not considered a satisfactory method in this multivariate analysis. Nevertheless, this reliability coefficient was calculated and described for a randomly selected number of stations (at station level and item level). A multiple regression method was used to measure the variability and distribution of test scores among the different stations, circuits, and master phases (second or third). This procedure was performed using the PROC MIXED statement in SAS. Finally, the borderline regression method was used to test the reliability of the scoring system.

## Results

**Figure 1.**
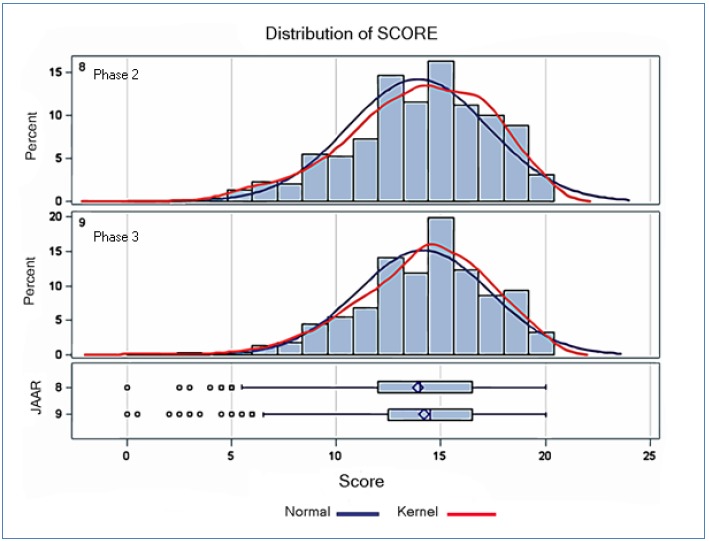
Distribution of test scores/20 per master phase (n=340)

**Figure 2.**
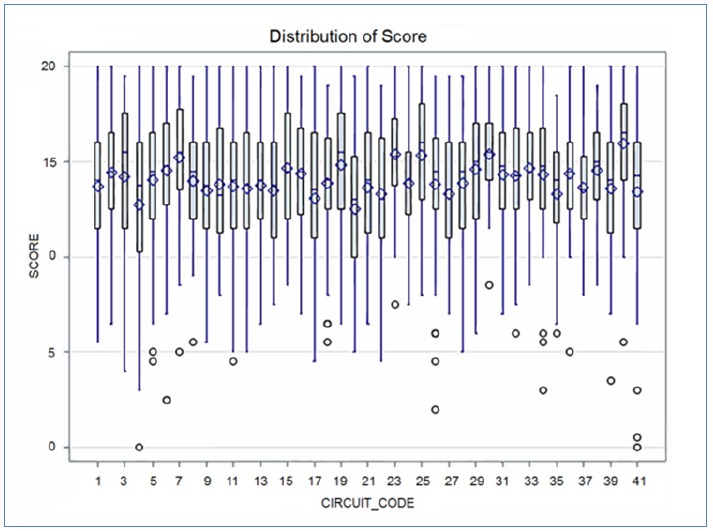
Distribution of test scores by circuit (n=340)

**Figure 3.**
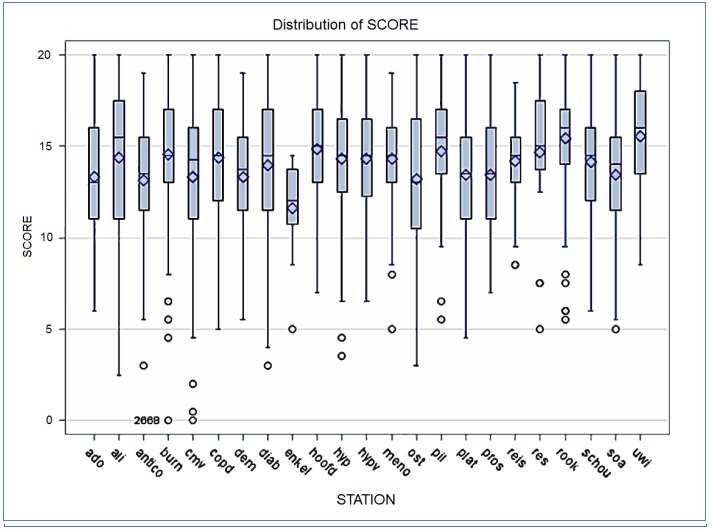
Distribution of test score by station (n=340)

Of the 32 newly developed stations, 24 were distributed over 40 different circuits. Eight stations were not included because they were considered inappropriate or did not meet the quality requirements after revision by the expert group (see METHODS). The features of the students are presented in Table 1. A total of 340 students participated, with second-phase students slightly overrepresented (57%). Ten students in the second master phase and four students in the third phase failed. Average final scores were slightly higher for third-phase students (t-test, p = 0.05). The score distribution did not differ significantly between the two master phases (Figure 1). Table 2 presents the results of the score distribution on both station level and circuit level. Although there was a significant difference in test scores for several circuits and stations, the overall score distribution remained stable and comparable. Variance and distribution of test scores per station and per circuit are presented in Figures 2 and 3, respectively. A multiple regression analysis revealed that test scores were dependent on the stations and circuits, but not on the master phase (Table 3).

**Table 1.** Students’ features per phase (n =340)

Characteristics	Second phase	Third phase
N Students	194 (57%)	146 (43%)
N Passed (standard setting overall test score > 11/20 and pass on >=4 stations)	184 (94%)	142 (97%)
Average test score /20 (SD)	13.9 (3.4)	14.2 (3.2)
T-Test of test scores	p < 0.05	
Median test score /20	14.1	14.5

Standards were set using the borderline regression method. Table 4 presents the results of a regression analysis with the overall test scores and the observer scores for phases 2 and 3. No significant difference was observed between the two scoring systems. Cronbach’s alpha was calculated to test reliability across stations, and was ≥0.8. An intra-station (on the item level) Cronbach’s alpha was found to fluctuate between 0.6 and >0.8 depending on the station. An intra-circuit Cronbach’s alpha showed levels between 0.7 and 0.85.

**Table 2.** Distribution of scores in between circuits and in between stations (Tukey’s studentized range) (n=340)

Source	Mean	r2	Coeff. var.	Mean square error	F	p
Circuit	14.0	0.05	23.0	10.5	3.28	0.001
Station	14.0	0.04	23.0	10.4	5.58	0.001

## Discussion

In this pilot study, a new format of the OSCE was tested in response to an increase of test scores with preserved psychometric qualities over the years. The test qualities of a more complex OSCE were studied. Students in the second and third phase of the GP master were assessed with an OSCE that consisted of eight 12-minute stations. Average test scores returned to acceptable results (14/20 or 70%), with a performance-based cut-off score of 11/20 and an overall failure rate of 5%. Overall test scores were equally distributed at station, circuit, and phase levels. The variance of score per station and per circuit on the overall test score was significant. Although Cronbach’s alpha was not with- held as an appropriate test method, values appeared acceptable to good at inter-station, inter-circuit, and item levels.

**Table 3.** Multivariate analysis with ‘score’ as independent variable (n=340)

Tests of fixed effects		
Effect	F	p
Station	8.62	<0.0001
Phase	1.17	0.28
Circuit	2.24	<0.0001
Circuit × station	3.35	<0.0001

In past years, students’ test scores on the traditional OSCE rose in the Flemish Interuniversity GP Master. Psychometric reliability and validity remained stable but content reliability failed. Consequently, a team of experts, staff members, and students participated in a discussion in search of an acceptable explanation. First, the past OSCEs were revised. In the junior-GP postgraduate education program, an OSCE is scheduled in the second and third phase of the GP master. For 15 years, a circuit of 20 8- minute stations was conducted following the ‘traditional’ and well-described method. The simulated patients were highly experienced and intensively trained, and they adopted various roles. Observers were recruited from the pool of internship supervisors. They were all experienced in work- place-based assessment and they were trained before enrollment in the OSCE. The blueprint of the OSCE was framed in agreement with the qualification requirements of the target students. Validity and reliability testing was performed after each session, and standard setting was performance-based (borderline regression method). Test qualities were systematically found to range from good to very good. Over the years, the OSCE was not subject to context changes.

Second, the literature was searched thoroughly. Reviews and meta-analyses regarding the test qualities of OSCEs concluded that reliability as expressed by Cronbach’s alpha of overall test scores is only moderately acceptable (0.60 or lower).^4,6,16,17^ An increase in examination time, an increased number of stations, and assessment by more than one observer were proposed as promoters of test quality. On the other hand, scores on some subscales of the OSCE (particularly addressing technical skills) appear to be more reliable than scores on other scales (e.g., communication).^18^ Even the design (context) of the OSCE was, for unclear reasons, recognized as a moderator of the estimated reliability 6. The fact remains that validity testing of an OSCE is a hazardous piece of work: content validity, construct validity, and concurrent validity testing are subject to many moderating and uncontrollable factors.^4^

Finally, a team of experts discussed the above findings. Since the benefits of OSCE testing are always precariously balanced against cost, the construction and content of this assessment concept require full attention.^19-21^ The intervention of a validation committee guarantees that a standardized and objective methodology is used to develop an OSCE. The content is determined and monitored by the learning objectives.^22^ In contrast the specificity of the OSCE design is barely addressed in curricula development and in research. Aside from a consensus on the number of stations and examination time, no other regulations are defined.^16^ Traditionally, each simulated patient encounter focuses on one particular theme, discloses one single announcement (e.g., complaint, symptom, or question) and targets one main instruction. In the light of a rapidly changing educational environment, this approach might underestimate the learners’ competences and skills. In the case of the GP master described in this study, it was concluded that the original OSCE was not sophisticated or complex enough to adequately assess the performance of future GPs.

**Table 4.** Logistic regression of item-checklist score and observer score (n=340)

Variable	Parameter estimate	*t*	p
Observer score Phase 2	0.5	1.4	0.17
Observer score Phase 3	0.4	2.0	0.06

Therefore, the traditional OSCE was extensively reshaped (see METHODS). Each patient encounter was initiated for more than one reason, and therefore focused on more than one theme and provided instruction on more than one competence. Because the changes were intended to increase the complexity and sophistication of the stations without increasing their difficulty, no pitfalls, rare diseases, complex medical problems, or other confounders were included. The scenarios were constructed similar to encounters in daily practice.

The distribution of the overall scores, as well as the distribution of the scores per station, per circuit, and per phase, demonstrated that the revised OSCE is reliable from a psychometric point of view. Although conventional reliability testing was deemed inappropriate (and therefore only conducted for a random sample of stations and circuits),

Cronbach’s alpha was moderate to high. This statistical approach was not considered because it addresses single component facets and does not address the overall normality of distribution.^17^ In the OSCE described in this pilot study, stations and circuits varied substantially. This structure required a multivariate approach rather than a correlation analysis.

The content and concurrent validity were not specifically addressed. However, median and mean scores appeared comparable over the different score levels (circuits, stations). Second, the significant concordance between the item score and the observer score is an argument in favor of content reliability. All observers were highly experienced in assessment and in education, and they were blinded to the weighting of each item on the itemized checklist. They referred the performance of each candidate to the target student that they teach and train in practice.

This study has some important limitations. Twenty-four new stations were developed and tested in 40 different circuits. Each circuit was composed according to the formal blueprint of learning objectives. A more homogenous construction of the circuits certainly would have benefited the power of the study results and facilitated the analytic process. Consequently, inter-rater reliability was not reproduced in a reliable way, since the number of stations per circuit was low. Only a limited number of stations were observed by two separate observers. The results were reassuring, but not robust enough to report. Second, one major barrier in the implementation of an OSCE is the price tag. It is obvious that a circuit of eight 12-minute OSCE stations is far less expensive than a traditional OSCE. Evidently, the burden of all participants must also be taken into the final account. In this study, feasibility, acceptability, and cost-benefit analyses were not addressed.

The first strength of this pilot study is that it was initiated and elaborated by a focus group consisting of experts, staff members, and students. Advice was also sought outside the faculty, and the final version of the test was presented to the panel members again. Second, both observers and simulated patients were recruited from the core pool. All were highly experienced and well instructed on the new OSCE design. Third, the results were very reassuring. As hypothesized, mean and median scores returned to acceptable values with preserved test qualities.

## Conclusions

In a changing learning environment, assessment and evaluation strategies require reorientation. The OSCE is considered a reliable tool to assess the clinical and vocation- al competencies of medical students. However, its reliability and validity remain subject to discussion. In particular, when it comes to content and design, the traditional OSCE might underestimate the performance level of postgraduate trainees in general practice. A reshaping of this OSCE to a more sophisticated design with more complex patient encounters appears to restore the validity of the test results.

Future research should repeat this pilot study to confirm test results, refine the concept, and define optimal test conditions.
